# NSUN2 relies on ALYREF to regulate Nrf2-mediated oxidative stress and alleviate Dox-induced liver injury

**DOI:** 10.1186/s13062-024-00477-y

**Published:** 2024-04-29

**Authors:** Yingying Huang, Xiao Li, Lin Wei, Shinan Ma, Liming Ma, Yuxin Zan, Xiju He, Yijun Tang, Yan Ding

**Affiliations:** 1 Hubei Key Laboratory of Embryonic Stem Cell Research, Hubei Provincial Clinical Research Center for Umbilical Cord Blood Hematopoietic Stem Cells, Taihe Hospital, Hubei University of Medicine, Shiyan, 442000 Hubei China; 2 Department of Pulmonary and Critical Care Medicine, Taihe Hospital, Hubei University of Medicine, Shiyan, Hubei People’s Republic of China

**Keywords:** Liver injury, NSUN2, ALYREF, m5C, Nrf2

## Abstract

**Background:**

Doxorubicin (Dox) is associated with various liver injuries, limiting its clinical utility. This study investigates whether NSUN2 participates in Dox-induced liver injury and the associated molecular mechanism.

**Methods:**

In vivo and in vitro liver cell injury models were constructed based on Dox therapy. The protein levels of NSUN2 and oxidative stress indicators Nrf2, HO-1, and NQO1 were evaluated by Western blot. The RNA binding potential was detected by RNA methylation immunoprecipitation (RIP). Additionally, the effect of NSUN2 on Nrf2 mRNA synthesis and localization was evaluated using an RNA fluorescence probe.

**Results:**

NSUN2 was downregulated, and liver tissue suffered significant pathological damage in the Dox group. The levels of ALT and AST significantly increased. NSUN2 interference exacerbated Dox-induced liver cell damage, which was reversed by NSUN2 overexpression. RIP demonstrated that NSUN2 recognized and bound to Nrf2 mRNA. Western blot analysis showed the protein level of Nrf2 in the NSUN2-WT group was significantly higher than that of the control group, whereas there was no significant change in Nrf2 level in the mutant NSUN2 group. Luciferase analysis demonstrated that NSUN2 could recognize and activate the Nrf2 5′UTR region of LO2 cells. In addition, RIP analysis revealed that ALYREF could recognize and bind to Nrf2 mRNA and that ALYREF controls the regulatory effect of NSUN2 on Nrf2.

**Conclusion:**

NSUN2 regulates Dox-induced liver cell damage by increasing Nrf2 mRNA m5C methylation to inhibit inhibiting antioxidant stress. The regulatory effect of NSUN2 on Nrf2 depends on ALYREF.

## Introduction

Doxorubicin, a widely used anti-tumor drug in clinical practice, is associated with drug-induced liver injury as one of the most prevalent adverse reactions. Long-term use of doxorubicin may pose severe negative effects on normal tissues, limiting its clinical application [1]. The degree of drug-induced liver injury is manifested as acute, subacute, or chronic, with severity varying from asymptomatic liver enzymes to fulminant liver failure and death. Therefore, understanding the mechanism of drug-induced liver injury is critical for clinical practice.

The mechanism of doxorubicin-induced hepatotoxicity is complex, with several systems at work. Recent studies have revealed that oxidative stress play an impartment role in Dox-induced hepatotoxicity [2]. Post-transcriptional RNA modification has been linked to the regulation of oxidative stress. 5-methylcytosine (m5C) is a post-transcriptional RNA modification in various RNAs, including mRNA, rRNA, tRNA, enhancer RNA, and non-coding RNA [3]. The regulators of m5C include methyltransferases (writers), demethylases (erasers), and methylated RNA-binding proteins (Readers). Among common m5C Writers are members of the NSUN family (NOP2, NSUN2-7) and the DNMT family (DNMT1, DNMT2, DNMT3A, and DNMT3B) [4], which mainly regulate the formation of RNA m5C modifications. Common m5C erasers include TET family proteins (TET1, TET2, and TET3) and ALKB dioxygenase family proteins (ALKBH1) [5, 6], which are primarily remove RNA m5C modifications. Common m5C readers include ALYREF, YBX1, and RAD52 [7, 8], which are primarily read RNA after m5C modification. m5C modifications can alter the molecular functions of mRNA such as pre-processing, splicing, nuclear export, mRNA translation, and stability, and are synergistically controlled by “writers,” “erasers,” and “readers” [9–11].

NSUN2 (NOP2/Sun RNA methyltransferase family member 2) is one of the most important RNA m5C methyltransferases. It catalyzes the formation of RNA m5C, which affects the stability of RNA and protein synthesis within cells, and thus plays a role in regulating several life processes such as cell proliferation, differentiation, and aging. Current research has found that NSUN2 was highly expressed in various types of tumors, including hepatocellular carcinoma, and regulate tumorigenesis and immunotherapy resistance [12]. In addition, a recent study showed that NSUN2 exerted anti-viral effects [13]. This is the first research to investigate the role of NSUN2 in drug-induced liver injury.

## Materials and methods

### Animal feeding and Dox induced liver injury model building

Male C57BL/6J mice (20 ± 2 g) were derived from the Laboratory Animal Center of Hubei University of Medicine (SYXK (Liao): 2013-0006). Mice in the 12 h of light and dark cycle, free access to food and water, with free access to food and water. The Animal Care and Ethics Committee of Hubei university of medicine approved the study. The treatment protocol complied with the ‘Guidelines for the Care and Use of Experimental Animals’ issued by the National Institutes of Health of China. C57BL/6J mice were injected with shNC or shNSUN2AAV9 virus through the tail vein to inhibit NSUN2 expression. One month later, shNC or shNSUN2AAV9 mice were randomly divided into control and DOX group. The Dox group received an intraperitoneal injection of 20 mg/kg Dox, while the control group received corresponding volume of physiological saline with the same injection method. The mice were sacrificed on days 1, 4, and 6 after Dox treatment to collect liver tissue for histological staining and Western blot analysis. Blood samples were collected from the mice on days 1, 4, and 6 for liver function tests (Table 1).

**Table 1:** Primer information

Gene	Primer sequence
Nrf2-F	5′TCA GCG ACG GAA AGA GTA TGA3′
Nrf2-R	5′CCA CTG GTT TCT GAC TGG ATG3′
GAPDH-F	5′AAC CGA GGA TGA CCA CAG TCG3′
GAPDH-R	5′TTG GCT CCT ACT GGT GTC AGC3′
NSUN2 sgRNA-1	TACAGCTGCGGATTGCAACACGCGGGGCTG
NSUN2 sgRNA-2	CCAGGAGCTCAAGATCGTGCCCGAGGGCGA
NSUN2 sgRNA-3	TACCTGCTCGTCCATCAAGCCAAGAGGCTG
ALYREF sgRNA-1	GGCGGCTGTGCACTATGATCGCTCTGGTCG
ALYREF sgRNA-2	TTGAGCGGAAGGCAGATGCCCTGAAGGCCA
ALYREF sgRNA-3	GCGTGGAGACAGGTGGGAAACTGCTGGTGT

### Extraction of primary hepatocytes

8-week-old mice were anesthetized by intraperitoneal injection of 1% sodium pentobarbital and sterilized with alcohol. The abdominal cavity was opened to locate the hepatic portal vein and inferior vena cava. A fine indwelling needle was inserted into the hepatic portal vein and fixed with a vascular clamp. Next, perfusion of Buffer I (Table 2) was initiated through a peristaltic pump (5 ml/min). The inferior vena cava was opened until no blood flew from the liver. Perfusion was continued with collagenase IV while the inferior vena cava was clamped with a hemostatic clamp until the liver tissue became soft. The liver was removed and placed into Buffer II (Table 3). The liver tissue was crushed using tweezers, filtered through a 100 µm mesh, and the filtered hepatocytes were collected into a 50 mL centrifuge tube. DMEM medium (Hyclone, sh30021.01) was added and gently mixed to resuspend the cells. The cells were centrifuged at 500 rpm for 2 min, and the supernatant was discarded. The pellet was resuspended in 20 mL of DMEM medium and centrifuged four times. The supernatant was discarded, and the pellet was resuspended in DMEM medium containing 10% fetal bovine serum (CellMax, SA101.02). The cells were then cultured in a 37 °C incubator with 5% CO_2_.

**Table 2:** Perfusion fluid (buffer I) (200 mL)

Reagent name	Dosage
HBSS buffer	200 mL
EDTA	0.5 mM

**Table 3:** Digestive juice (buffer II) (200 mL)

Reagent name	Dosage
HEPES	715 mg
low-sugar DMEM	200 mL
Collagenase IV	100 mg

### Liver function testing

Mouse blood ALT (C009-1-1) and AST (C010-2-1) tests were assessed using the test kit and following the instructions of the Nanjing Jiancheng Bioengineering Institute. Mouse serum was used as the measurement group and physiological saline was used as the control group. 5 µl of serum or physiological saline was added to a 96-well plate, with 3 replicate wells in each group. 20 µl of ALT/AST substrate solution was added to each well, and incubated at 37 ℃ for 30 min. Subsequently, 20 µl of 2, 4-dinitrophenylhydrazine was added and incubated at 37 ℃ for 20 min. Finally, 200 µl of 0.4 mol/L NaOH was added to each well, and left to stand at room temperature for 10 min. The absorbance value was measured at 505 nm wavelength, and the concentration of ALT/AST was calculated.

### TUNEL staining of liver tissue

Apoptosis in liver tissue cells was evaluated using the one-step TUNEL cell apoptosis detection kit (Beyotime, C1089). After staining, add DAPI to stain the nucleus at room temperature (RT) for 10 min, then seal with anti-fluorescence quenching sealing solution, and pictures were taken under a fluorescence microscope (Olymplus, IX53 + DP73).

### Immunohistochemical staining

Immunohistochemical detection was performed according to our previous method [14]. liver Tissue samples were dewaxed, underwent antigen retrieval treatment, and sealed. They incubated with Rabbit NSUN2 (Proteintech, 20,854-1-AP, 1:500) or Nrf2 (Proteintech, 16,396-1-AP, 1:200) polyclonal antibodies at 4 °C overnight, and then with goat anti-rabbit second antibody (ZSGOBIO, PV-9001, 1:200) at room temperature for 30 min. Finally, the results were tested by using the DAB Chromogenic Kit (ZSGO-BIO, ZLI-9018).

### Western blot

The total protein of liver cells or tissue was extracted and separated on sodium dodecyl sulfate–polyacrylamide gel electrophoresis (SDS-PAGE). Next, the proteins were transferred onto the PVDF membrane, and incubated with rabbit NSUN2 (Proteintech, 20,854-1-AP, 1:1000), Nrf2 (Proteintech, 16,396-1-AP, 1:1000), ALYREF (Abclonal, A6010, 1:1000), HO-1 (Beyotime, AF1333, 1:1000), NQO1 (Beyotime, AF7614, 1:1000), GAPDH (Beyotime, AF1186, 1:2000), and α-Tubulin Rabbit polyclonal antibody (Beyotime, AF5012, 1:2000), were overnight at 4 °C. The Horseradish peroxidase (HRP) labeled goat anti-rabbit IgG (H + L) (Beyotime, A0208, 1:2000) was added and incubated at room temperature for 1 h. Finally, the concentration of the proteins was measured using the enhanced chemiluminescence (ECL) chemiluminescence kit (Beyotime, P0018).

### Flow cytometry detection of cell apoptosis

Cell apoptosis was detected by using the Annexin V-FITC Cell Apoptosis Detection Kit (Beyotime, C1062L). The human non-tumor hepatic cell line LO2 cells [15] (a generous gift from Dr Huangtian Yang, the Chinese Academy of Sciences, Shanghai, China) were cultured in DMEM medium (Hyclone, sh30022.01) contain 10% FBS (CellMax, SA101.02), and digested with 0.05% trypsin. Next, 200 µL Annexin V-FITC mixture were added to 1 × 10^5^ cells suspension. Subsequently, 10 µL of propidium iodide staining solution was added and the mixture was incubated at RT for 20 min. Finally, cell apoptosis in each group was detected by flow cytometry.

### NSUN2 and ALYREF CRISPR/Cas9 targeting

LO2 cells in the logarithmic growth phase were plated into 6-well plates. When they reached a confluence of 40–50%, 10µL vehicles or NSUN2 or ALYREF CRISPR/Cas9 lentivirus was added. After 48 h, cells were treated with puromycin to screen cells with successful targeting. The sgRNA sequences for NSUN2 and ALYREF are presented in Table 1.

### CCK8 assay

LO2 cells were treated with 0, 0.5, 1, 2, 3, 5, 10 μmol/L Dox in vitro for 24 h, after that CCK8 assay kit (Beyotime, C0038) was used to detect cell viability. Three replicate wells in each group.

### Flow cytometry detection of ROS

The level of cell ROS was assessed using the Reactive Oxygen Specifications Assay Kit (Beyotime, S0033S) as directed by the manufacturer. LO2 cells were cultured for 24 h, and 1 × 10^6^ cell suspension was treated with 2 μmol/L Dox for 12 h. Following that, 500 mL of serum-free DMEM containing DCFH-DA (1:1000) was added and incubated at 37 ℃ for 30 min. The cells were collected and the fluorescence intensity was examined by flow cytometry.

### RNA immunoprecipitation (RIP)

RNA immunoprecipitation assay was conducted according to our previous method [14]. Briefly, LO2 cells were irradiated under 1500J UV crosslinker for cross-linking, then lysed with RIPA lysis buffer on ice, and the supernatant was collected. 100 µl of the supernatant was assigned to the input group, and the remaining solution was divided into two equal parts. One group was subjected to ALYREF/NSUN2-RIP, and the other group was subjected to IgG-RIP. 10µL protein A&G magnetic beads were pipetted, incubated on a shaking bed at 4 ℃ for 1 h, then centrifuged at 4 ℃ and 12,000 rpm for 1 min, and the supernatant was discarded, keeping the precipitate. Based on the experimental design, one group was treated with either ALYREF or NSUN2 antibody, while the other served as a control and was treated with IgG antibody. Both groups were then incubated on a shaking bed at 4 ℃ overnight. Following this, 10 µL of protein A&G agarose magnetic beads were introduced and subjected to centrifugation at 4 ℃ and 12,000 rpm for 1 min, after which the supernatant was discarded, retaining the precipitate. Subsequently, each tube underwent 5 washes with 500 µL of RIP Wash Buffer to ensure thorough cleansing. RNA extraction was carried out by adding 1 ml of Trizol to each tube. The RNA extracted from both the experimental and control groups, as well as from the Input group, was utilized for RT-PCR analysis. The primer sequences employed in this study are provided in Table 1.

### RNA fluorescence in situ hybridization

This assay employed a sensitivity-enhanced in situ hybridization detection kit V (MK1034, Boster, China) following the instructions. LO2 cells in the logarithmic growth phase were cultured for 24 h, then fixed at RT with 4% paraformaldehyde for 30 min, digested with 3% pepsin at room temperature for 10 s, and incubated in 50 µl pre-hybridization solution at 37 ℃ for 2 h. The digoxin-labeled Nrf2 mRNA probes (AXYBIO, CDS-HO8845-19, 1 µg/ml) were added to the cells, incubated overnight at 37 ℃, and closed with a close solution at 37 ℃ for 30 min. The cells were then incubated with biotinylated mouse anti-digoxin at 37 ℃ for 1 h and with SABC-FITC at 37 ℃ for 30 min. Finally, the tablets were sealed with a DAPI-containing anti-fluorescence quencher and examined under Olymplus laser confocal microscope (FV3000RS). The positive cells fluoresced yellow-green fluorescence, whereas the nucleus fluoresced blue.

### Firefly luciferase report

The active site of NSUN2 methyltransferase was revealed as Cysteine at 321 (321C) using protein structure analysis and database search. A mutant plasmid with C321A was then constructed (Cysteine at position 321 was mutated into alanine). In addition, we constructed Nrf2 CDS, 3′UTS and 5′UTS plasmids. LO2 cells were co-transfected with empty vector, NSUN2 wild (NSUN2 WT) or NSUN2 C321A (NSUN2 Mut) plasmids, and Nrf2 CDS, Nrf2 3′ UTS or Nrf2 5′UTS, respectively, using Lipofectamine 3000 (Invitrogen, L3000008). The cells were collected after 48 h and identified with a firefly luciferase report kit (YEASEN, 11402ES60). The plasmid and the virus used in this study were constructed and synthesized by WZ Biosciences Inc.

### Statistical analysis

The data was analyzed using Prism-8.02 software and presented as mean ± standard deviation (SD). The unpaired t-test was used to compare the two groups. Differences were deemed statistically significant at *P* < 0.05.

## Results

### NSUN2 participates in regulating Dox-induced liver injury

**Fig. 1:**
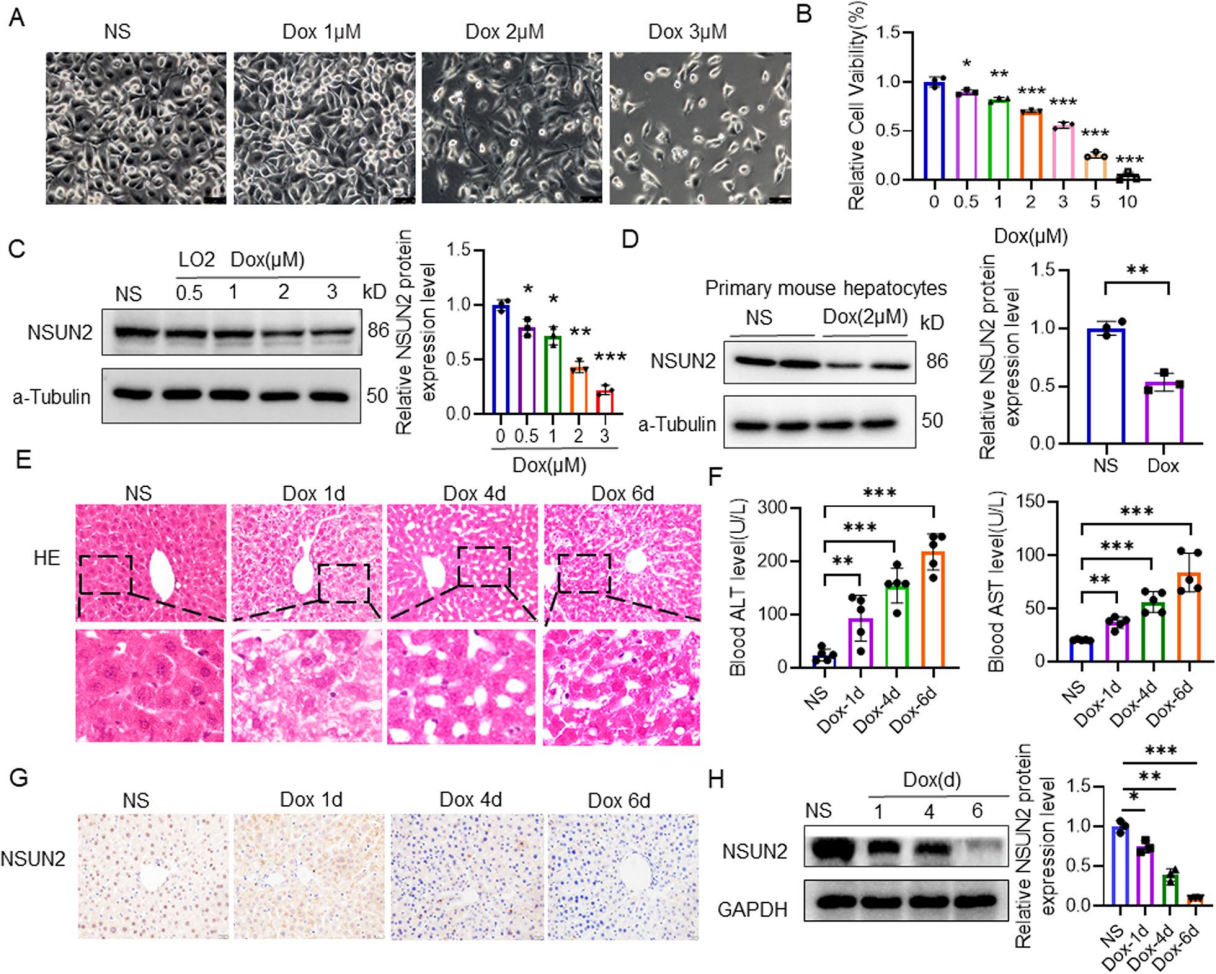
Dox downregulates NSUN2 levels in liver cells. **a** The morphology of LO2 cells after 24 h of Dox treatment (n = 3, scale bar = 75 µm). **b** The effect of Dox treatment on LO2 cell vitality (n = 3). **c** Western detection of the effect of Dox treatment on NSUN2 expression in LO2 cells (n = 3). **d** Western detection of the effect of Dox treatment on the expression of NSUN2 in primary liver cells (n = 3). **e** Representative images of liver tissue HE staining in the mice (1, 4, and 6 day) under a microscope (n = 3, scale bar = 20 µm). **f** Serological detection of ALT and AST (n = 5). **g** Immunohistochemical detection of liver NSUN2 expression (n = 3, scale bar = 20 µm). **h **Western detection of NSUN2 expression in liver tissue (n = 3). The data is represented by the average ± SD of three trials, and compared with the control group, *P < 0.05, **P < 0.01, ***P < 0.001

Phase-contrast microscopy revealed different degrees of cell death after treatment (Fig. 1a). CCK-8 assay showed that the cell viability of Dox group decreased compared with the control group, and the cell survival rate of 2.0 μmol/L Dox group was 70.1% (Fig. 1b). The cells were treated with 2.0 μmol/L Dox in the subsequent steps. Western blot results revealed that the level of NSUN2 protein in the Dox group decreased in a concentration-dose dependent manner (Fig. 1c). In addition, assessment of mouse liver primary cells showed that NSUN2 protein levels were decreased significantly after 2.0 μmol/L Dox treatment for 24 h (Fig. 1d).

HE staining revealed that hepatocytes in the Dox group displayed vacuolization, atrophy, disordered arrangement of hepatocyte cords, and cell necrosis as compared with the control group (Fig. 1e). Moreover, the degree of liver damage worsened over time. The blood ALT and AST levels in the Dox group were significantly higher compared with the control group (Fig. 1f). Immunohistochemical and Western detection revealed that the protein level of NSUN2 in the liver tissues of the Dox group was significantly lower than in the control group, and the expression level of NSUN2 decreased as liver damage increased (Fig. 1g, h).

### Interference with NSUN2 expression promotes Dox-induced liver injury

**Fig. 2:**
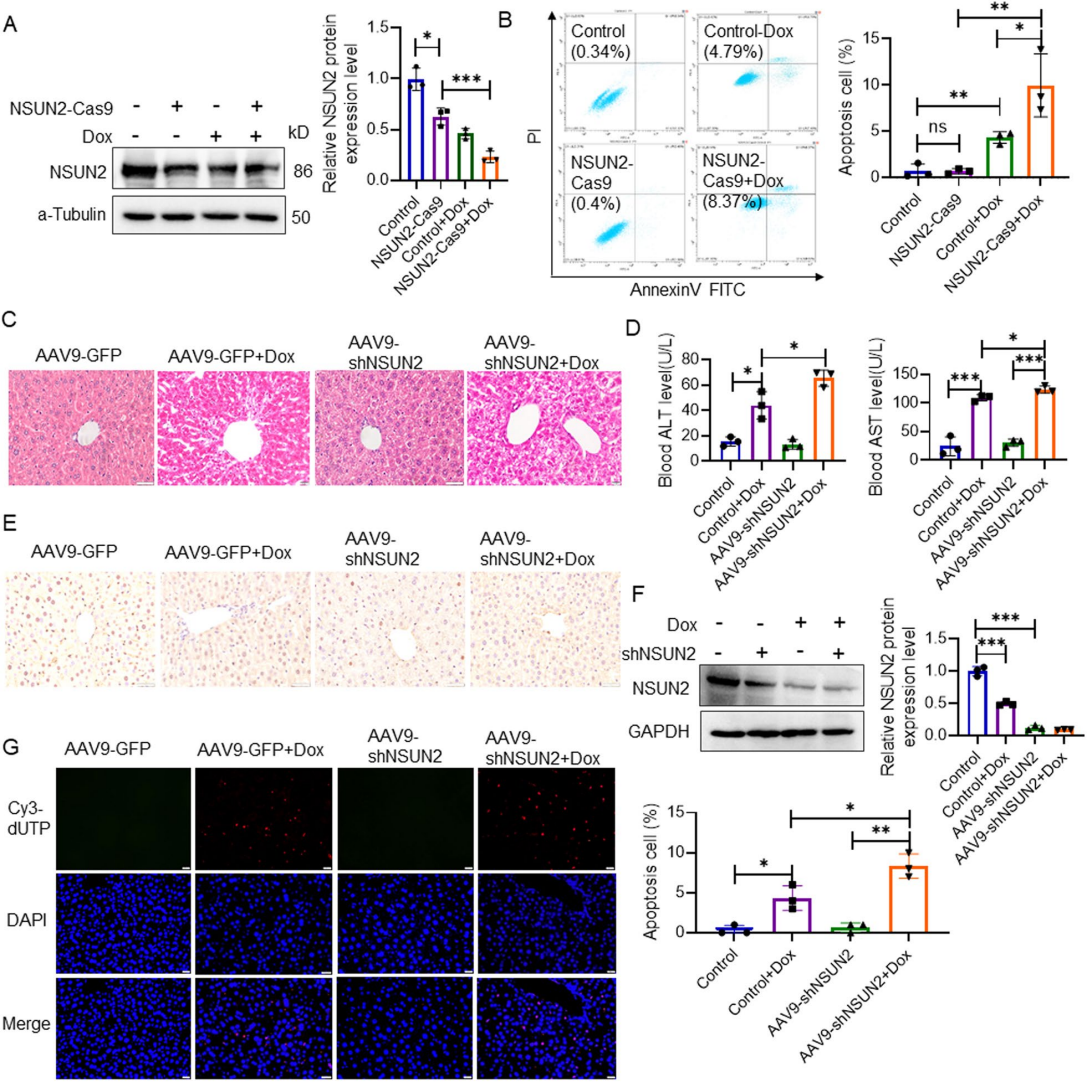
NSUN2 knockout promotes Dox-induced LO2 cell damage. **a** Western blot detection of the expression of NSUN2 in LO2 cells after Dox-induced injury (n = 3). **b** Flow cytometry detection of the apoptosis level of LO2 cells following Dox-induced injury (n = 3). **c** HE detection of pathological changes in 3 days after Dox-induced liver injury (n = 3, scale bar = 20 µm). **d** Serological detection of ALT and AST in 3 days after Dox-induced liver injury (n = 6). **e** Immunohistochemical detection of NSUN2 expression in the liver of AAV9-shNSUN2 liver injury mice (n = 3, scale bar = 20 µm). **f** Western blot detection of NSUN2 expression in liver tissues of each group in 3 days after Dox-induced liver injury (n = 3). **g** TUNNEL detection of liver apoptosis level in shNSUN2 liver injury mice (n = 3, scale bar = 20 µm). The data is represented by the average ± SD of three trials, and compared with the control group, *P < 0.05, **P < 0.01, ***P < 0.001

Western blot analysis showed that the expression of NSUN2 was significantly decreased in the NSUN2-Cas9 group compared with the control group (Fig. 2a). Flow cytometry revealed that the apoptosis rate of the NSUN2-Cas9 + Dox group was significantly higher than that of the control group (Fig. 2b).

HE staining analysis revealed that the AAV9-shNSUN2 + Dox group exhibited increased liver pathological damage compared with the AAV9-GFP + Dox group (Fig. 2c), and blood ALT and AST levels were significantly higher than the AAV9-GFP + Dox group (Fig. 2d). Western and immunohistochemical tests revealed a significant decrease in NSUN2 expression level in the AAV9-shNSUN2 group (Fig. 2e, f). TUNEL detection demonstrated that the AAV9-shNSUN2 + Dox group had more positive cells with red fluorescence compared with the AAV9-GFP + Dox group (Fig. 2g).

### NSUN2 overexpression alleviates Dox-induced liver injury

**Fig. 3:**
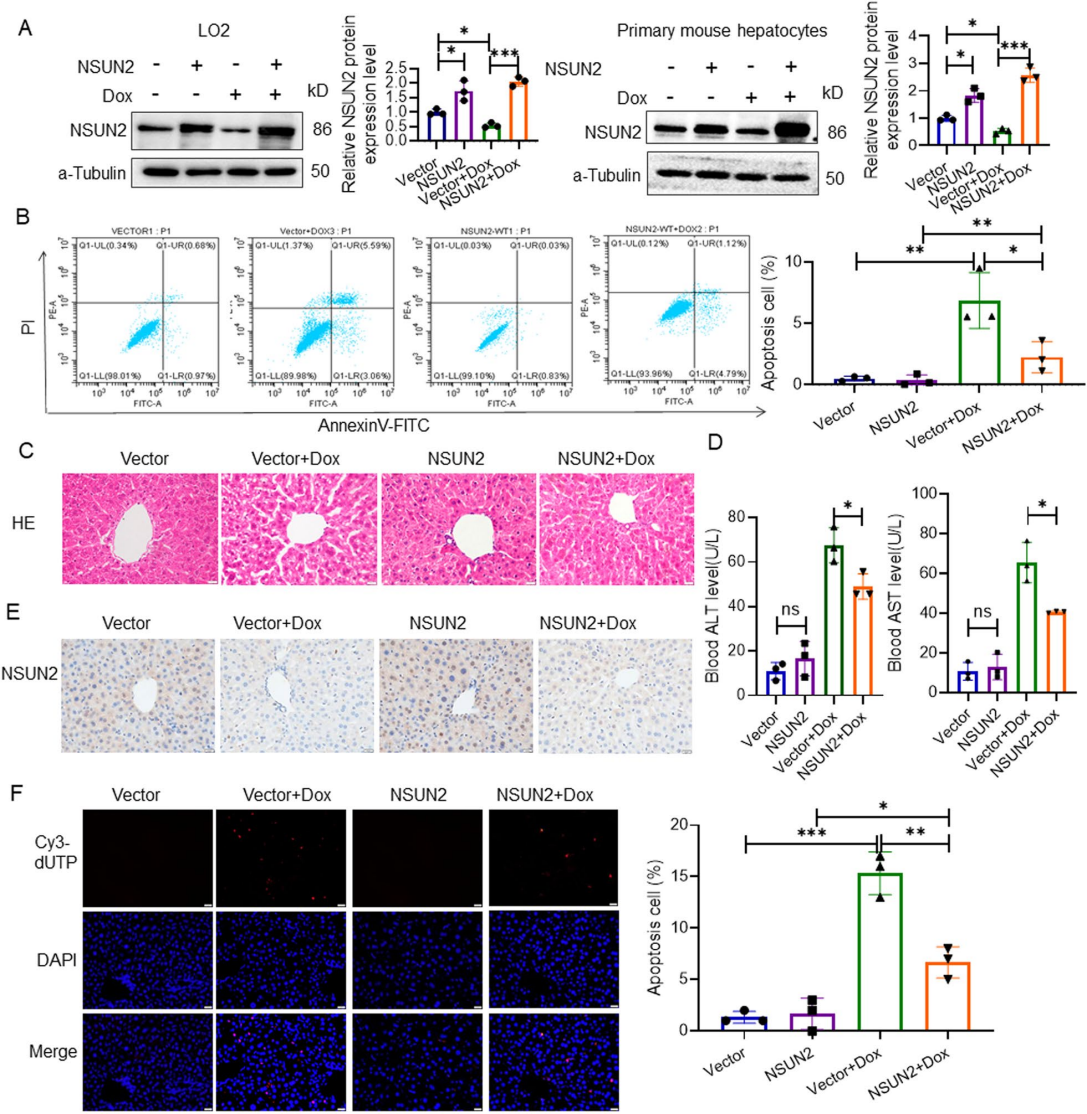
Overexpression of NSUN2 alleviates Dox-induced liver damage. **a** Western detection of NSUN2 expression in LO2 and liver primary cells after Dox treatment (n = 3). **b** Flow cytometry detection of the level of apoptosis in LO2 cells overexpressing NSUN2 following Dox-induced injury (n = 3). **c** HE detection of pathological changes in Dox-induced liver injury in AAV9-NSUN2 mice (n = 3, scale bar = 20 µm). **d** Serological detection of ALT and AST in 3 days after Dox treatmen (n = 3). **e** Immunohistochemical detection of NSUN2 expression in the liver of AAV9 AdNSUN2 liver injury mice 3 days after Dox treatment (n = 3, scale bar = 20 µm). **f** TUNEL detection of apoptosis cells in AAV9-AdNSUN2 liver injury mice in 3 days after Dox treatment (n = 3, scale bar = 20 µm). The data is represented by the average ± SD of three trials, and compared with the control group, *P < 0.05, **P < 0.01, ***P < 0.001

LO2 cells and primary liver cells of mice were infected with lentivirus to induceNSUN2 overexpression, then treated with Dox treatment to induce cell damage. Western blot results revealed that the expression of NSUN2 in AdNSUN2 cells was higher compared to the GFP group (Fig. 3a). Flow cytometry showed that the apoptosis rate of LO2 cells in the NSUN2 + Dox group significantly decreased compared with the Vector + Dox group (Fig. 3b).

HE staining analysis revealed that the AAV9-NSUN2 + Dox group exhibited decreased liver pathological damage compared with the AAV9-GFP + Dox group (Fig. 3c). The plasma ALT and AST levels of AAV9 AdNSUN2 + Dox group mice were significantly lower compared with the AAV9 GFP + Dox group (Fig. 3d). Immunohistochemical analysis revealed a significant increase in NSUN2 expression level in the AAV9-NSUN2 group (Fig. 3e). TUNEL detection revealed that the AAV9-NSUN2 + Dox group had significantly fewer positive cells with red fluorescence compared with the AAV9-GFP + Dox group (Fig. 3f).

### NSUN2 alleviates Dox-induced liver injury through Nrf2-mediated antioxidant stress response

**Fig. 4:**
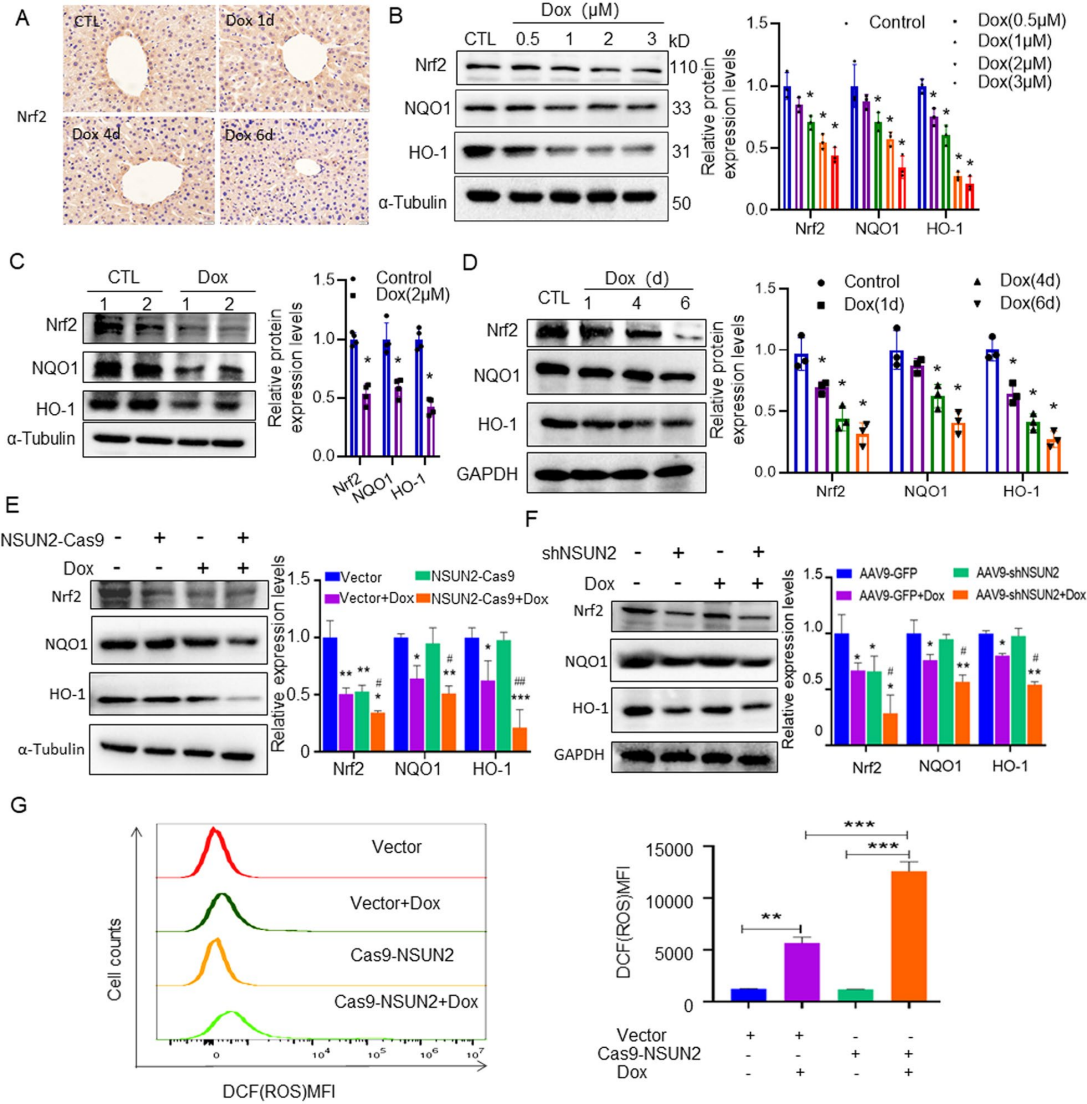
NSUN2 downregulates Nrf2 and promotes Dox-induced oxidative stress response in liver cells. **a** Immunohistochemical staining detected Nrf2 expression in the liver of mice treated with saline or Dox (n = 3, scale bar = 20 µm). **b** Western detection of Nrf2, NQO1, and HO-1 expression in LO2 cells treated with different concentrations of Dox (n = 3). **c** Western detection of Nrf2, NQO1, and HO-1 expression in liver primary cells after Dox treatment (n = 3). **d** Western detection of Nrf2, NQO1, and HO-1 expression in liver tissues after 1, 4 and 6 days of Dox treatment (n = 3). **e** Western detection of Nrf2, NQO1, and HO-1 expression in WT or NSUN2-Cas9 LO2 cells treated with 2 µM of Dox. **f** Western detection of Nrf2, NQO1, and HO-1 expression in control or shNSUN2 mice liver tissues 3 days after 20 mg/kg Dox treatment (n = 3). **g** Flow cytometry detection of NSUN2 knockdown on ROS levels in LO2 cells. The data is represented by the average ± SD of three trials, and compared to the control group, *P < 0.05, **P < 0.01, ***P < 0.001. Compared to vector Dox group, ^#^*P* < 0.05, ^##^*P* < 0.01

In vitro experimental analysis demonstrated that Dox treatment significantly decreased the protein levels of Nrf2, HO-1, and NQO1 with concentration and dose dependent manner (Fig. 4a). The levels of Nrf2, HO-1, and NQO1 proteins in primary liver cells significantly decreased after 24 h of treatment with 2 mol/L Dox (Fig. 4b). In vivo experiments revealed that the expression level of Nrf2 in the liver tissue in the Dox group was significantly reduced compared with the control group (Fig. 4c). Western blot results revealed that Nrf2, HO-1 and NQO1 protein levels showed a decreasing trend in the liver tissue of Dox mice during the culture period compared to the control group (Fig. 4d). Furthermore, Western blot analysis demonstrated that the expression of Nrf2, HO-1, and NQO1 in the NSUN2-CAS9 + Dox group was significantly down-regulated compared with that in the Vector + Dox group after 24 h of treatment (Fig. 4e, f). ROS detection revealed that the ROS level in the NSUN2-Cas9 + Dox group was significantly higher than in the control + Dox group (Fig. 4g).

### Regulatory effect of NSUN2 methyltransferase activity on Nrf2

**Fig. 5:**
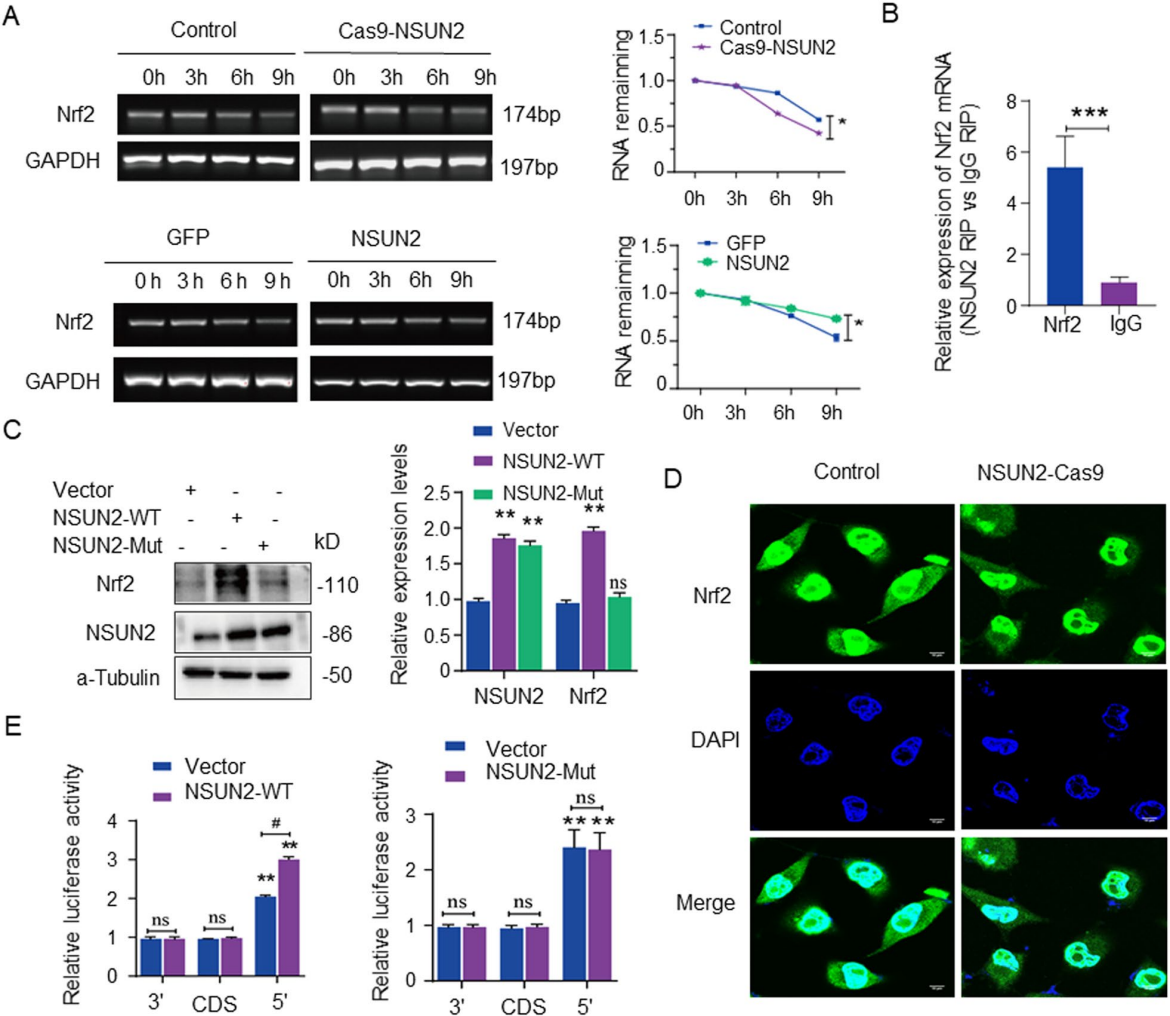
Effect of NSUN2 on Nrf2 mRNA. **a** RT-PCR detection the half-life of Nrf2 mRNA in LO2 cells of each group after actinomycin treatment (n = 3). **b** RIP detection the combination and recognition ability of NSUN2 and Nrf2 mRNA (n = 3). **c** Western blot analysis of the effect of NSUN2 wild-type or mutant plasmids on Nrf2 protein level (n = 3). **d** RNA fluorescence probe detection of the effect of NSUN2 overexpression on Nrf2 mRNA (n = 3, scale bar = 10 µm). **e** Firefly luciferase detection of the binding region between NSUN2 and Nrf2 (n = 3). The data is represented by the average ± SD of three trials, and compared with the control group, *P < 0.05, **P < 0.01, ***P < 0.001

We investigated whether NSUN2 influences the protein expression of Nrf2 by regulating its mRNA modification. RT-PCR analysis revealed that the half-life of Nrf2 mRNA in the NSUN2-Cas9 group was significantly shortened after actinomycin treatment compared with the control group, and the half-life of Nrf2 mRNA in the NSUN2 group was prolonged significantly (Fig. 5a) Besides, RIP-PCR analysis revealed that NSUN2 recognized and bound to Nrf2 mRNA (Fig. 5b).

Furthermore, a mutant plasmid for the putative site of NSUN2 methyltransferase was constructed and transfected into LO2 cells. Western blot analysis demonstrated that the NSUN2 protein levels in the NSUN2-WT and NSUN2-Mut groups increased compared with the empty vehicle. Meanwhile, as compared with the vehicle group, the protein level of Nrf2 in the NSUN2-WT group was significantly higher, but the NSUN2-Mut group exhibited no significant change (Fig. 5c). The probe detection of Nrf2 mRNA revealed that the fluorescence intensity of Nrf2 mRNA in the nucleus and cytoplasm of the NSUN2-Cas9 group was significantly lower than that of the vehicle group (Fig. 5d).

Nrf2 pGL3-5 ′UTR, pGL3-CDS, and pGL3-3′ UTR plasmids were constructed and co-transfected with the vehicle, NSUN2-WT, or NSUN2-Mut plasmid, respectively. Firefly luciferase experiments demonstrated that the pGL3-5′ UTR group had the highest luciferase count compared with the pGL3-CDS and pGL3-3 ′UTR groups. The overexpression of NSUN2-WT and pGL3-5 ‘UTR groups had significantly higher luciferase values compared with the vehicle group. There was no significant difference in luciferase values of the NSUN2-Mut and pGL3-5 ′UTR group compared with the vehicle group (Fig. 5e).

### NSUN2 regulates ALYREF-dependent Nrf2 protein expression

**Fig. 6:**
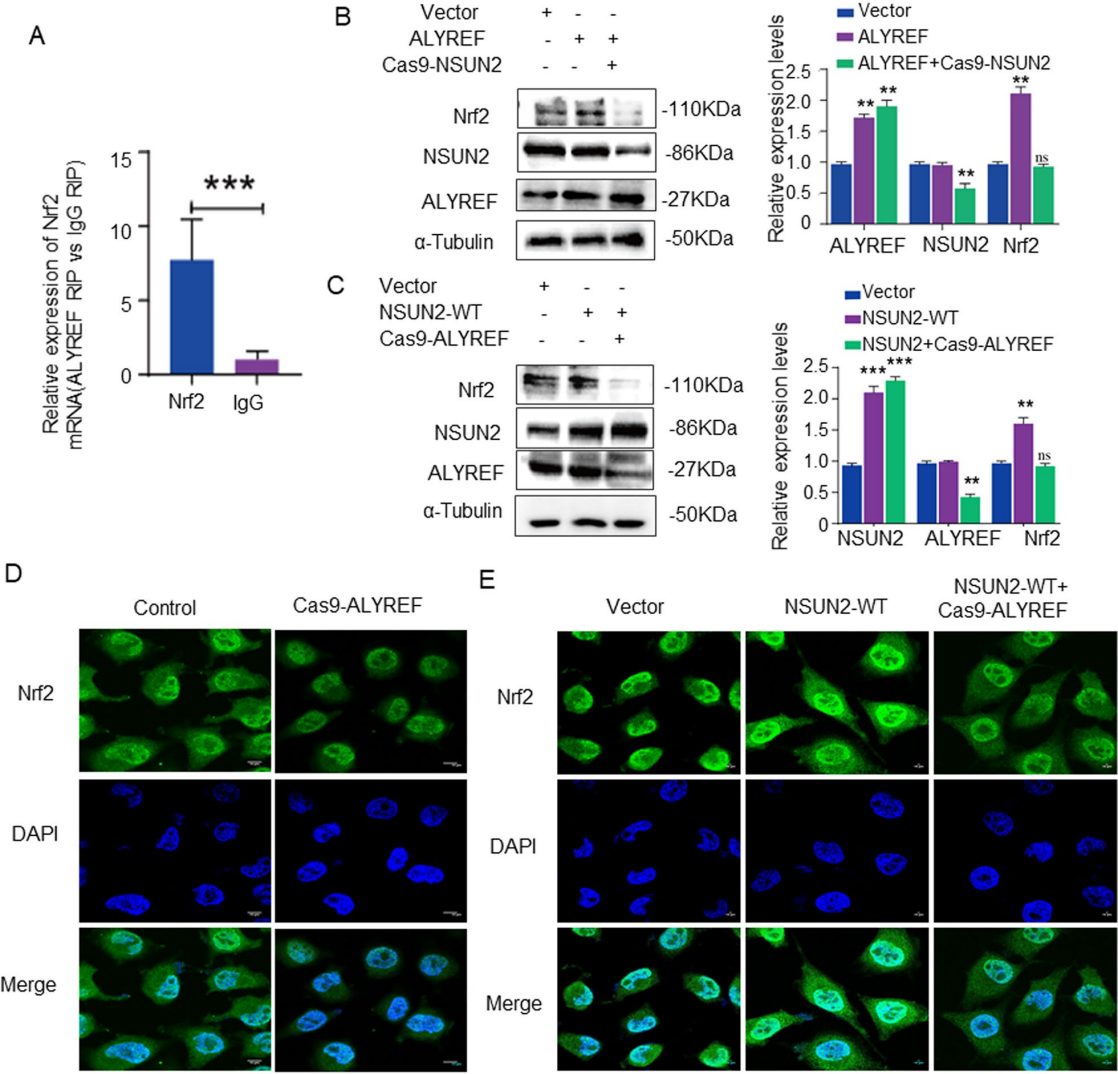
ALYREF recognizes and binds Nrf2 mRNA. **a** RIP detection of the combination and recognition ability of ALYREF and Nrf2 mRNA (n = 3). **b** Western detection of NSUN2, Nrf2, and ALYREF protein expression in each group (n = 3). **c** Western blot detection of the expression of NSUN2, Nrf2, and ALYREF proteins in each group (n = 3). **d** RNA fluorescence probe detection of the effect of ALYREF-Cas9 on Nrf2 mRNA (n = 3, scale bar = 10 µm). **e** RNA fluorescence probe detection of the effect of ALYREF-Cas9 and NSUN2 overexpression on Nrf2 mRNA (n = 3, scale bar = 10 µm). The data is represented by the average ± SD of three trials, and compared with the control group, *P < 0.05, **P < 0.01, ***P < 0.001

RIP-PCR analysis revealed that ALYREF recognized and bound to Nrf2 mRNA (Fig. 6a). The vector control or ALYREF overexpression plasmid and NSUN2-Cas9 plasmid were co-transfected into LO2 cells. Western blot analysis revealed that the expression level of ALYREF protein in the ALYREF overexpression group increased significantly compared with the vector group; besides, the expression of NSUN2 protein did not change significantly, while the expression of Nrf2 protein increased significantly. The ALYREF WT + NSUN2 Cas9 group showed no significant change in the expression level of ALYREF protein when compared with the vector group, while the expression level of NSUN2 protein decreased. The expression level of the Nrf2 protein exhibited no significant change (Fig. 6b).

NSUN2-WT or vehicle was co-transfected with ALYREF-Cas9 plasmid into LO2 cells. Western blot analysis revealed a significant increase in NSUN2 and Nrf2 protein levels when compared to the vector (vehicle) group (*P* < 0.05). However, there was no significant change observed in ALYREF levels. When compared to the NSUN2-WT group, significant decreases were observed in the protein levels of ALYREF and Nrf2 (*P* < 0.05), whereas NSUN2 protein levels remained unchanged in the NSUN2-WT + ALYREF-Cas9 group (Fig. 6c).

In situ hybridization detection of the Nrf2 mRNA probe revealed that the fluorescence intensity of Nrf2 mRNA in the nucleus and cytoplasm of the AYLREF-Cas9 group was significantly decreased compared with the control group (Fig. 6d). Notably, AYLREF-Cas9 attenuated the increased fluorescence intensity of Nrf2 mRNA in the nucleus and cytoplasm caused by NSUN2 overexpression (Fig. 6e).

## Discussion

The liver is the primary site for drug metabolism in the body. While several drugs have preventive and therapeutic effects on the body, they also potentially trigger toxic reactions during the biological transformation process of the liver, which can cause fatal fulminant liver failure. Dox is a clinical anticancer drug limited by severe toxic side effects [16]. The current study investigated the role and mechanism of Dox in liver injury. We demonstrated through experiments that NSUN2 expression is downregulated in a Dox-induced liver injury model. NSUN2 interference promoted Dox-induced liver injury, whereas NSUN2 overexpression alleviated Dox-induced liver function increase, and inhibited liver cell apoptosis, and damage. These data imply that NSUN2 is a potential therapeutic target for Dox-induced liver injury.

Dox induced hepatotoxicity mechanism is complex, there are several system at work. Recent research has revealed that oxidative stress is a critical mechanism of Dox-induced hepatotoxicity. Nrf2 (nuclear factor eroid-2 related factor 2, Nrf2) is an oxidation–reduction dependent transcription factor that regulates the expression of major antioxidant and detoxifying enzymes [17]. In particular, heme oxygenase-1 (HO-1) is a phase II enzyme that regulates heme metabolism and is the rate-limiting step for the production of various antioxidant intermediates [18]. Mounting evidence emphasizes the role of HO-1 in protecting the liver and kidneys from oxidative damage and inflammation. In addition, literature reports indicate that zedoary turmeric ketone reduces oxidative stress, prevents mitochondrial damage, activates the Nrf2/HO-1 signaling pathway, and alleviates Dox-induced activation of extracellular signal-regulated kinase 1/2 (Erk1/2) and c-Jun N-terminal kinase (JNK) [19]. Natural compounds have also been demonstrated to inhibit oxidative stress, mitochondrial dysfunction, and cell apoptosis by upregulating the Nrf2/HO-1 signaling pathway, hence preventing Dox-induced cardiotoxicity [20, 21]. In the present study, we discovered that, compared with the control Dox treatment group, the NSUN2 interference Dox treatment group significantly increased oxidative stress response in the interference NSUN2 expression model, while Nrf2 and its downstream HO-1, NQO1 proteins were significantly downregulated. Meanwhile, in the overexpression NSUN2 expression model, the NSUN2 overexpression Dox treatment group significantly decreased oxidative stress response compared with the control Dox treatment group, and the oxidative stress-related protein Nrf2 and its downstream HO-1 and NQO1 proteins were significantly upregulated. These data fully demonstrate that NSUN2 overexpression increases Nrf2 expression to protect the liver from oxidative stress damage.

NSUN2 wild-type and methyltransferase key site mutant plasmids were constructed to examine the mechanism of NSUN2 regulation of Nrf2. We discovered that the NSUN2 overexpression plasmid significantly increased Nrf2 protein expression compared to the control group following transfection into LO2 cells. Cells with NSUN2 mutant plasmids exhibited no significant difference in Nrf2 protein expression. RNA methylation immunoprecipitation verified the binding of NSUN2 to Nrf2 mRNA. Recent studies indicate that m5C modification primarily occurs in the 5’ UTR and 3’ UTR of mRNA, with significant peaks in the translation initiation codon [22, 23]. Firefly luciferase analysis revealed that the luciferase values in the 5’ UTR group were significantly upregulated, and compared with the control, the luciferase values were significantly upregulated in the overexpression NSUN2 5‘UTR group, while there was no significant difference in the luciferase values in the mutant NSUN2 5’ UTR group. These findings demonstrate that m5C mediates NSUN2 methyltransferase-driven regulation of the protein expression of Nrf2.

The synthesis of m5C in mRNA is mainly catalyzed by the RNA methyltransferase NSUN2. ALYREF specifically recognizes extracellular transport of m5C mRNA and is the main recognition protein of m5C. Previous studies have demonstrated that ALYREF and Y-box binding protein 1 (YBX1) recognize and bind to the m5C motif, playing a role in the occurrence and progression of tumors [24]. NSUN2-mediated m5C methylation promotes the outflow of ATX mRNA from the nucleus to the cytoplasm in an ALYREF-dependent manner, driving cell motility [25, 26]. In the present study, RIP analysis revealed that ALYREF also recognizes and binds Nrf2 mRNA. The expression of Nrf2 protein in LO2 cells increased significantly after transfection with NSUN2 virus, but NSUN2 over-expression and ALYREF knockdown did not significantly influence the protein expression level of Nrf2. ALYREF overexpression in LO2 cells significantly increased the expression of Nrf2 protein, while the effect of NSUN2 knockdown and ALYREF overexpression on the protein expression level of Nrf2 was influence insignificant. These results demonstrate that ALYREF is required for the regulation of NSUN2 during Nrf2 protein expression.

**Fig. 7:**
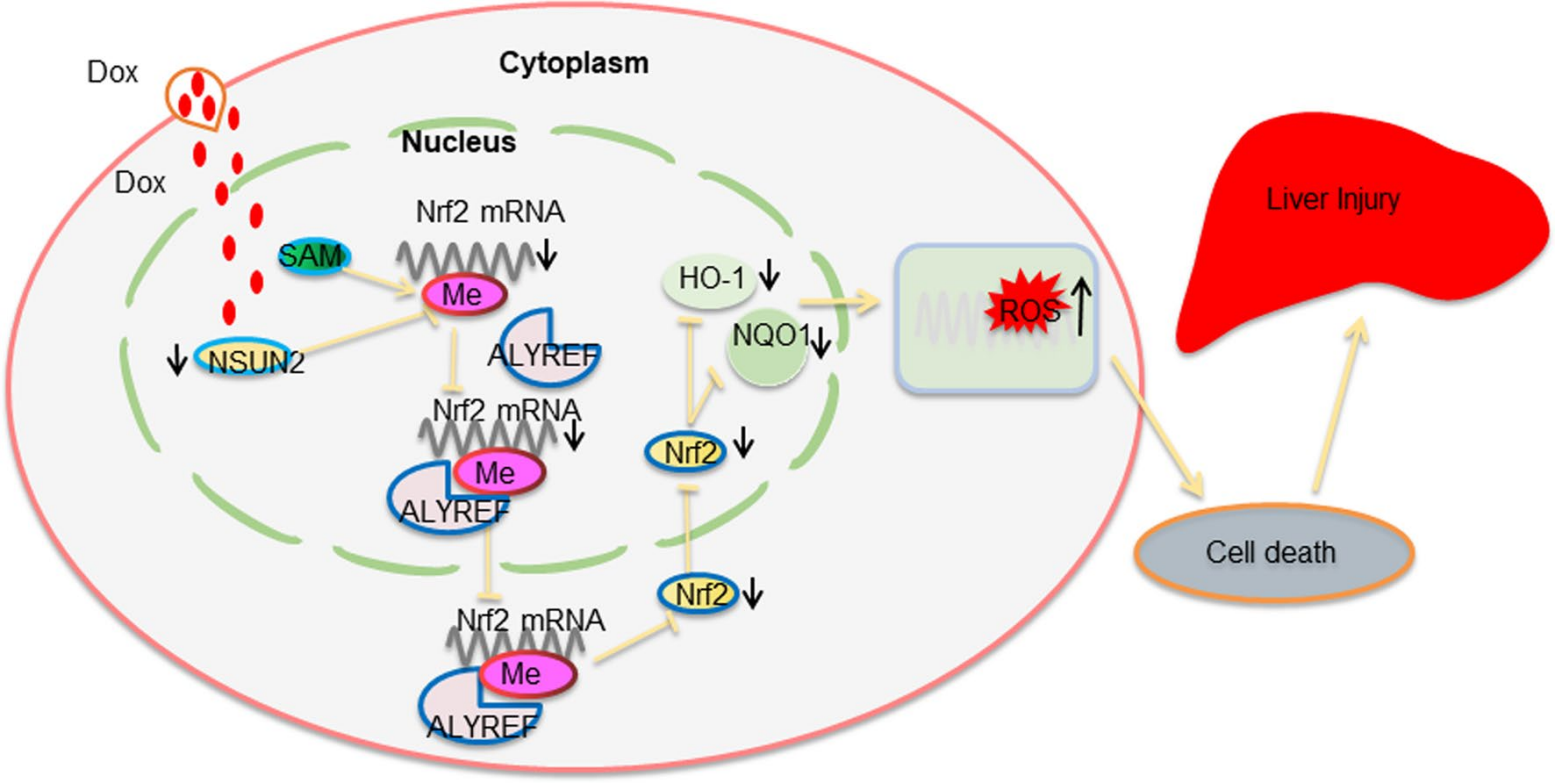
Scheme of NSUN2 in DOX-induced liver injury

In conclusion, this study reveals that NSUN2 expression is decreased in DOX-induced liver injury and that overexpressing NSUN2 can mitigate DOX-induced liver injury. Meanwhile, NSUN2 promotes ALYREF-dependent methylation modification of Nrf2 m5C mRNA, promotes its protein expression, and activates the Nrf2-mediated antioxidant stress response, thereby alleviating DOX-induced liver cell apoptosis (Fig. 7).

## Data Availability

All the data used during the study are available from the corresponding author on request.
